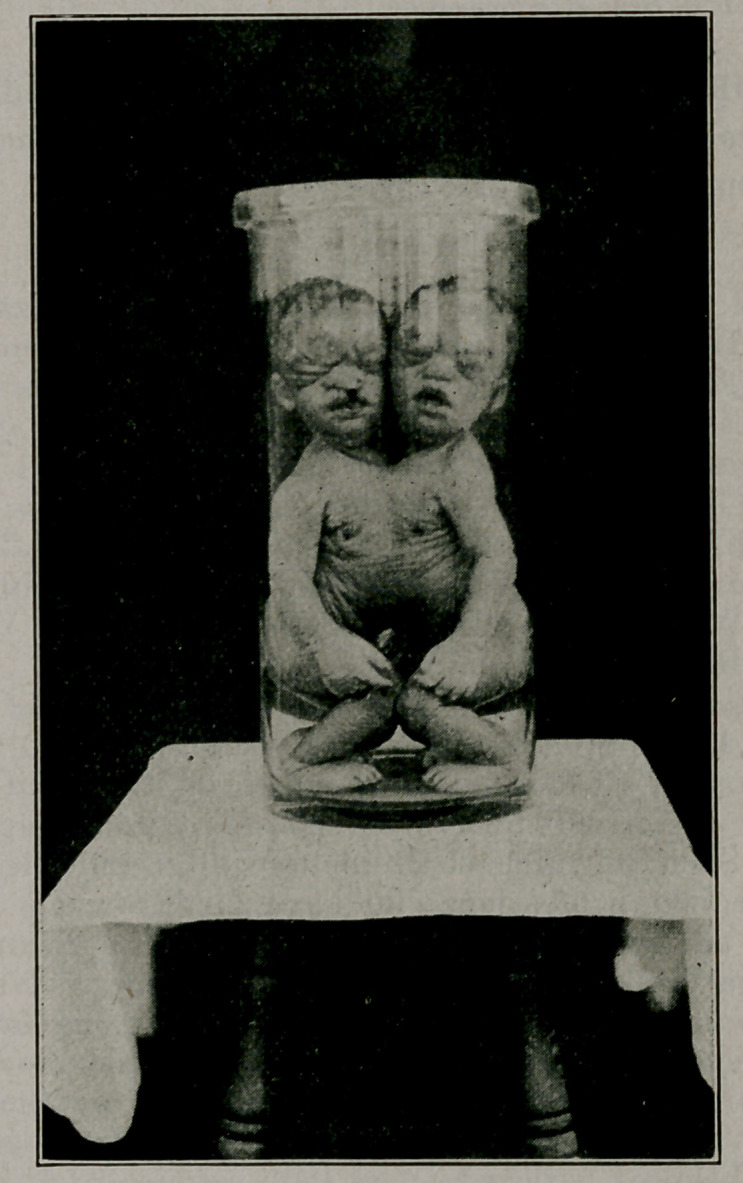# Topics of Public Interest

**Published:** 1916-09

**Authors:** 


### TOPICS OF PUBLIC INTEREST

---

**State Road Fatality.** During the last week of July an accident occurred on the state road at Irving, N. Y. that seems to us to raise an issue very different from the ordinary run of automobile accidents due to congestion or individual carelessness. On account of repairs to a small bridge or culvert traffic was detoured to the south side of the regular road for a few feet, with a dip of four or five feet below the road level. The temporary roadway was, as usual in such circumstances, rough and clayey. After regaining the level of the highway, a space of about 20 feet intervened before a trolley crossing and, for traffic toward Buffalo, this crossing was almost entirely hidden both by the higher level of the roadway at the side of the detour and by a rank growth of weeds between the culvert and the trolley track. A Pennsylvania car driven by a woman, after dark, in the rain, was struck by a trolley bound toward Erie, carried about a hundred feet along the track, demolished and the driver killed. In dry weather, by day-light, knowing the exact location of the trolley track and the exact condition of the detour, we found that it was easy enough to negotiate the detour on low speed and to come to a full stop before reaching the trolley track, although none too much space was available. Even a careful driver, not fully acquainted with the exact permanent and temporary conditions, driving at minimum high-gear speed, or going into neutral, or changing to second speed to meet an emergency could not give attention to the bad detour and see a trolley approaching; could not maintain a speed sufficient to avoid stalling in the mud on the sharp upgrade and stop in time to avoid an approaching trolley if suddenly warned by another occupant of the car. The highway mentioned is not only a state road but an interstate highway, largely used by persons unfamiliar with exact local conditions. A more skillfully constructed death trap could scarcely have been designed for military purposes, to intercept an enemy car. Primarily on those in charge of the construction and ultimately on the state, rests the responsibility for this death and the other injuries inflicted—in a moral if not a legal sense.

---

**The Stevens Pure Food Bill Pending in Congress** has been endorsed by the Retail Grocers' Assn. of N. Y. State.

---

**Exclusion of Canadian Nurses.** Nurses from Ottawa, engaged on account of the demands imposed by the epidemic of infantile paralysis by hospitals in New York City, were turned back at Alburg, Vt., being dragged from their berths at night and put out into the rain. Immigration officials claim that they were acting under the alien labor law and that no unnecessary hardship was imposed. We have never been able to comprehend why an immigrant should be required to be able to show that he was not liable to become a public charge and, at the same time, should be compelled to trust to luck to get employment in this country. In the present, instance, the nurses were not immigrants and their employment was a matter of urgent need on the part of this country, a matter of personal devotion to professional duty on their part and, in a sense, a matter of international courtesy on the part of their country. It is most unfortunate that anything tending toward unpleasant international feeling should have occurred. When will we learn the lesson that the power to pass a law should be in accordance with a right on the part of the government, to make reasonable exceptions to it and comprehend the general principle that reasonable discretion should be granted to anyone empowered to inforce a law or rule?

---

**State Quarantine Against Rabies** has been threatened in the case of Buffalo. A number of dog bites have been reported, and 26 dogs' brains have shown Negri Costics. The local regulations as to muzzling of all dogs are being inforced rigorously and the killing of unlicensed dogs is proceeding as rapidly as possible. We understand that Buffalo has had 1 human death from rabies in its entire history—14 years ago. It is said that most dispensaries treat more bites by human beings than by dogs and it is a matter of common observation that a large share of dog bites are due to molestation by the persons bit. All scientific reports show that rabies is more common in cold than in warm weather and we do not understand why dog laws should not be sensibly inforced throughout the year. Meantime, the local health and police authorities seem to be perfectly competent to control the situation without state intervention.

---

**Anterior Poliomyelitis** was first clinically described by von Heine, a German, in 1840.

---

**Workingmen and Alcohol.** A recent study of how one thousand workingmen spend their spare time and their spare cash indicates that by far the largest amount of spare cash is spent for beer—23 per cent of the total. Including beer, wine and whisky, the amount of spare cash spent by workingmen for intoxicating liquor amounts to 34 per cent of the total. These percentages are not of the total amount earned —they represent the items over and above what is required for the necessities of life.

The expenditure for beer shows a steady decline as wages increase—for example, men earning $35 or more per week spend 7.6 per cent of their spare cash for beer. Those receiving less than $10 per week spend 39.9 per cent of their spare cash for beer.

It has also been discovered that the man who works the longest hours per day spends the most time in the saloon. This proves conclusively that the workingman who is most fatigued at the end of the day's work is most likely to crave artificial stimulant.—Col. Med., Aug.

---

**Brief for Health Insurance.** From American Association for Labor Legislation, N. Y. City. A death rate for American wage earners twice that of professional men; the prevalency of high sickness rates; the need among workers of better medical care and of a systematic method of meeting the wage loss incident to sickness; and the necessity for more active work in the prevention of disease, are the corner stones of the case for compulsory health insurance presented in the brief just published in New York by the American Association for Labor Legislation. This situation, it is pointed out, cannot be met fully by existing agencies, and can only be properly remedied by a system of health insurance embracing all wage earners and dividing the cost among employee, employer and the state.

The great amount of sickness in the homes of the poor causes an average loss by each wage-earner of 9 days in the year, and involves annually a national wage loss of approximately $500,000,000. Notwithstanding the greater prevalency of tuberculosis among wage-earners, their early susceptibility to the degenerative diseases of middle life, and the excessive death rate among the industrial population, workers often are unable to secure the medical attention they require. In Rochester, N. Y., it was found that 39 per cent of the sickness cases were not under a doctor's supervision; in a city like Boston, Mass., one-fourth of the population, it is estimated, are unable to pay the fees of a private physician.

The lowered vitality and the poverty created by present day conditions it is claimed can only be checked by a system of health insurance, which for a small sum divided among employer, worker and state, will bring medical care to the wage-earner and his family, will assure for a maximum of 26 weeks in a year a weekly payment of two-thirds of wages during the bread-winner's illness, and in addition a small funeral benefit should he die. "Compulsory health insurance," concludes the brief, "is an economical means for providing adequately for the sick wage-earner, and will prove a mighty force for the inauguration of a comprehensive campaign for health conservation."

---

**Prize for Artificial Hand.** The National Society of Surgery of Paris, through the generosity of an anonymous donor, offers a prize of $50,000 francs for the best invention, which must come from a citizen of an allied or neutral nation. There must be presented to the society, a patient who has used the device for at least six months, and the society will experiment with apparatus offered in competition for time as it deems necessary. The competition will close two years after the conclusion of the war. The successful apparatus will remain the property of the inventor. Those interested should apply for full information to M. le Secretaire General de la Societe Nationale de Chirurgie, Paris, 12 rue de la Seine.

---

**Retired and Non-Practicing Physicians.** Especially in the preparation of obituaries, we are seriously hampered by the lack of available data regarding physicians who have retired from practice. For statistic purposes, if no other, it is desirable to have a reliable and complete list of such members of the profession. The list published by the state society is very meagre. It might well be supplemented with many names from the nominally active list, but that is a matter of individual choice. Will our readers who have abandoned the medical profession for other kinds of work or who know of such non-practicing physicians, supply information for extending this list?

**Father Jogues,** a Jesuit missionary of the seventeenth century has been named for canonization. He was killed by the Indians at Auriesville, N. Y. His description of the ethnology of the Iroquois is of great value and some of his notes, as his very modest tale of his previous tortures, have a direct bearing on historic medicine.

**Indian Centenarian.** Gwan-ha-day, the oldest Iroquois, died at Toledo, last month, at the age of 104, leaving a widow 98 years old. The remains were buried on the Salamanca Reservation.

**Increase in the Public Health Service.** An appropriation has been made for 33 additional Assistant Surgeons, the salary being $2,000, with perquisites and subject to increase by promotion. It is expected that examinations will be held every month or so until the requisite number of eligible candidates have been secured. Those interested should apply to the Surgeon General, U. S. Public Health Service, Washington, for invitation to be examined.

**Educational Statistics.** Critics of the A. M. A. should give proper credit to this organization and its executive officers for the very valuable statistics on professional education, annually published in the Journal. If the A. M. A. merely compiled the statistics and had done nothing to secure the improvements noted, and if its officials were guilty of all the sins of omission and commission charged against them, the Educational Number of the Jour. of the A. M. A. would alone constitute an important offset.

In 1916 there were 13,121 non-sectarian students, 638 homoeopathic and 263 eclectic, other destinations of colleges having ceased to exist in 1911. The total, 14,022 is 869 below that for 1915, and more than a thousand less than in 1890.

Graduates this year numbered 3,274 non-sectarian, 166 homoeopathic, 78 eclectic, total 3,518, 18 less than in 1915. There were only 134 women included, the percentage having remained about 4 for the last 12 years. 26.9% of all graduates held liberal arts degrees, a gradual increase from 15.3% in 1910.

There are now 95 medical schools conferring degrees, 53 having been merged and 41 abandoned since 1905, the maximum total being 162 in 1906. 99 colleges, however, conferred degrees in 1916, 68 being Class A, 18 B and 13 C. Of the students, however, 79.6% attended A, 14.9% B, and 5.5% C colleges.

24 states now require one year of college training and W. Va. will do so for the session of 1917-18. 8 of these will require two years within a few years as do 9 already.

It may be claimed that the educational problem for our profession has been solved, according to reasonable standards of both preliminary and professional training, to the degree of approximately 90%. The economic problem connected with it has been solved to the extent that, from 1913 to 1919 (the number of students assuring against any sudden influx of matriculants at least for this period) new graduates will not have exceeded deaths in the profession by more than 500 a year. So far as can be estimated, this excess will have been provided for directly, by newly established national, state and other governmental positions. At any rate, the population is growing at a greater rate than the profession. Of course, it will still require many years to produce an equilibrium between demand and supply.

The report contains a great deal of interesting information aside from the gross statistics quoted.

---

**Novocaine Excluded From Harrison Law.** In a suit by the Farbewerke-Hoechst Co. to recover taxes paid under protest, according to the ruling of the Internal Revenue Dept., the U. S. Court in N. Y., Judge Grubb presiding, has decided that novocaine does not come under the law. Apparently, the same principle has been demonstrated for holocain, orthoform and anaesthesin. A crious coincidence is that the president of the company, Herman A. Metz, was a member of congress who took an active part in securing the passage of the Harrison bill. This law should certainly be amended so as to prevent arbitrary decisions by persons untrained in medicine, contrary to fact and common sense. In the past, it has been ruled that, under certain circumstances personal consultation with a physician is not a personal consultation, as well as that drugs which are in no sense narcotics or habit-forming, are so.

---

**Bequests of the Late J. William White.** $150,000 endowment of a professorship of surgical research in the University of Pensylvania; $5,000 to the College of Physicians of Philadelphia (this organization being a medical society, not a teaching institution); $10,000 whose interest is to be divided into three equal parts and used, respectively, as a prize for nurses in the University Hospital, as a prize for internes in the same, and for the purchase of Christmas presents for children in the hospital wards. Be it remembered, that Dr. White forbade any religious service at his funeral, was not a religious man in any sense, yet this last little bequest—ample for the purpose—impresses us more deeply than the larger endowment. Could anything be more truly Christian?

---

**The Poliomyelitis Epidemic,** according to statistics issued by the U. S. Public Health Service Aug. 17, has invaded 38 states. 11,117 cases have been reported, many on farms or in small villages. The principal focus of the epidemic seems to be Brooklyn. Greater N. Y. City has reported 6,573 cases and the state, mainly near N. Y. City, about 1,000 more. 1,714 cases have been reported from N. J.; 336 from Pa.; 105 from Mass. N. H. had reported only 7 cases, in spite of thousands of visitors for the summer from the infected area, and in no case could contagion be traced. Among 30,000 children in state institutions, using the same water, milk and food supplies as private families in the metropolis, no cases had occurred. No case had developed near the N. Y. City garbage dumps. Even in Brooklyn, there was no excess of cases in crowded and poor sections of the city. Quite a few adult cases have occurred, as a woman, aged 20, at Curtis, Steuben Co., where 5 cases of the disease and 1 death have been reported up to Aug. 20; a divinity student aged 22 at Sherrill, near Utica; a second adult in Steuben Co.; Dr. Leroy Vail of Flushing, aged 30. A moderate number of cases have occurred, singly or two or three to a town, in western N. Y. Buffalo shows the lowest record for several years: 1910, 52 cases, 5 deaths; 1911, 9 cases, no death; 1912, 316 cases, 23 deaths; 1914, 9 cases, 2 deaths; 1915, 31 cases, 5 deaths; 1916, to Aug. 20, 2 cases, 1 death. Considerable inconvenience has been due to enforcement of quarantine, Pennsylvania rigorously excluding entrance of children without a health certificate. Dr. G. W. Goler, Health Commissioner of Rochester, having had two cases in N. Y. children provided with certificates has refused to issue anything more than a statement of actual number of cases (2 we understand to date) and absence of clinical symptoms. It is proposed to institute national quarantine regulations.

---

**Sewage Disposal Plant for Fredonia.** Low water in Canadaway Creek, into which sewage flows, has caused sufficient concentration to be objectionable. Steps have been taken toward a remonstrance to the State Board of Health with the hope of reviving a plan for the erection of a sewage disposal plant. A few weeks ago, we stopped on the outskirts of Fredonia for water for a radiator, and received some drawn from a spring in the bed of another creek, near the junction of two roads. The statement was volunteered that this water was much used for domestic purpose by neighbors, though usually boiled. There is practically no stream in western N. Y., except high up on hills, which can safely be used for drinking or other domestic purposes without boiling.

---

**Teutonic War Statistics.** Prisoners, 1914-15, 1,695,000; 1915-16, 2,658,000. Of German wounded, 90.2% returned to active service, 1.4% died, the remainder were permanently unfit for service.

---

**Opium Trade.** A Chinese from Niagara Falls, under treatment in the Buffalo Municipal Hospital as an opium addict, was sentenced Aug. 3 to six months in the penitentiary for smuggling opium into the hospital and selling it to patients, thus explaining the failure of treatment in several cases. Later it was claimed that he did not bring it into the hospital, but sold it to patients outside.

---

**Sunstroke.** 100 deaths of human beings, 50 among infants, are said to have occurred in Chicago in one day, toward the close of July. Hundreds of horses are said to have been killed by the heat. In Buffalo, the total number of deaths was 10, up to the beginning of relatively cool weather early in August. It occurs to us that the relation of sunstroke and heat prostration to temperature and humidity, and the very marked seasonal and local differences which do not seem to run parallel with these factors, should be very carefully studied, for the whole country. We cannot free ourselves from the conviction that sunstroke is largely due, though indirectly, to alcoholism, and that another important factor is unnecessarily heavy clothing. The class most liable to sunstroke, is in our experience, prone to wear woolen undergarments throughout the year.

---

**The New York State Civil Service Commission** calls attention to the numerous openings for physicians in state hospitals, prisons and charitable institutions. Salaries range from about $1200 to $2000. There is a lack of applicants or, at least of successful candidates for some positions. Those interested should address the commission at Albany.

---

**Automobile Statistics.** In 1906, 48,000 motor vehicles were registered in the U. S. During the year ending July 1, 1916, 2,445,664 were registered, about 1 to 44 inhabitants. As the millionth Model T Ford was turned out last winter and as the sales for the present season must more than balance the earlier Fords which have passed out of commission, Mr. Ford's expressed ambition two or three years ago to have every third car a Ford, must have been realized. In Alabama, there is only 1 car for every 200 persons while the maximum was reached in Iowa, with 1 for every 16, or about 1 car for every three families.

---

**Theft of Narcotics.** On the night of July 25-6, 180 cocaine tablets and a hypodermic syringe were stolen from the office of Dr. E. F. Burns of Buffalo.

---

**Steuben County Tuberculosis Hospital.** A site in the village of Addison has been favorably considered, the only other one regarded as available being about two miles from Canisteo. Residents of Addison are protesting against the selection of that village, though assured by local and other physicians of the absence of danger. (Note: At the risk of seeming to oppose progress, we can not quite reconcile the well established relation of the tubercle bacillus to the disease with the assurance that a collection of patients involves no danger to the immediately surrounding population. A tuberculosis hospital should be easily accessible by railroad but it would seem possible in all instances to secure a site sufficiently isolated from actual habitations to insure the adequacy of natural modes of disinfection, largely by sun light, within the time and space limits of danger from direct contact, dust, etc., and to avoid contamination of streams).

---

**Base Hospital Unit for Rochester.** Our Associate Editor, Dr. Chas. W. Hennington, is one of the prime movers in securing volunteers for the entire personnel of a base hospital, to be offered to the United States, at any time that it may be required.

---

**The Johnson Cottage,** a land mark of Buffalo, has recently been demolished. It was the residence of Dr. Ebenezer Johnson, first mayor of the city of Buffalo in 1832, and again mayor in 1834, and well known, before and after his mayoralty, as prominent physician and citizen. It was built in 1833-4. One of the three great disadvantages of this country, is its lack of antiquities—the other two being the high cost of living and the interference with personal liberty. The last two advantages of the western European nations are, of course, seriously interfered with by the war which is also leveling the first difference. One can almost count on his fingers the American cities in which there is any building worth looking at on account of age. If we continue to destroy buildings at 50 and 75 years, we shall have none a century old. The cut is furnished by courtesy of Mr. Frank H. Severance, Secretary of the Buffalo Historical Society, having been published in "The Picture Book of Earlier Buffalo," Vol. 16 of the Society's publications.

**Postponement of Amalgamation.** The University of Pennsylvania and Jefferson medical schools will conduct their courses independently during the coming session, in order to afford time for considering a number of details in regard to the merger.

---

**Reduction of Typhoid in Toronto.** In 1910, the typhoid mortality was 40.8 per 100,000 population. In 1915, it was 1.9, the lowest for any American city of 350,000 and over.

---

**Change of Name:** The International Health Commission of the Rockefeller Foundation, to International Health Board of the Rockefeller Foundation.

---

**War Casualties.** We have previously commented on the apparent exaggeration of fatalities, loss by wounds, prisoners, etc., in the European War. The following table published in the Literary Digest, for the first two years of the War, based on published lists and careful estimates is 1,700,000 lower for killed; less than a million greater for wounded and prisoners together, and about 800,000 lower on the aggregate losses than statements for the first ten months of the war. We are not certain, however, that the 2-year statistics include prisoners and, in the Civil War, "missing" usually meant dead but without identification of corpses. Even if this qualification applies to the present war, the killed, wounded and missing for the two years are only about 2 million in excess of the same estimates for the first ten months.

| | Killed | Wounded or Missing | Total Casualties |
|---|---|---|---|
| Germany | 907,327 | 2,255,300 | 3,162,627 |
| Austria-Hungary | 500,000 | 1,500,000 | 2,000,000 |
| Turkey | 60,000 | 240,000 | 300,000 |
| Bulgaria | 40,000 | 110,000 | 150,000 |
| France | 800,000 | 1,200,000 | 2,000,000 |
| Great Britain | 150,000 | 470,000 | 620,000 |
| Russia | 1,000,000 | 4,000,000 | 5,000,000 |
| Italy | 35,000 | 140,000 | 175,000 |
| Belgium | 30,000 | 120,000 | 150,000 |
| Total | 3,522,327 | 10,035,300 | 13,557,627 |

---

**Super-Medication.** Our Associate Editor, Dr. C. Haase of Elmira, has recently been consulted by a woman who states her previous medication (in writing) as follows: Tablets for kidneys (swamp root) every 4 hours; strychnine 1/60 gr. every 4 hours; tonic after meals; massage with oil of turpentine twice daily; asafoetida pills; powder after meals for indigestion; cascarets for constipation, after meals; cod liver oil, three times a day; dessertspoonful of Duffy's Malt Whiskey, four times daily; egg and milk twice daily; white pine syrup with tar, for the air passages; iron and wine as tonic.

---

**Joined Twins.** W. P. Durham of Sasser, Ga., Am. Jour. Clin. Med., Aug., reports a case similar to that of Dr. Laura

M. Plantz in the June issue. They were delivered without difficulty at the seventh labor of a negress, stillborn, total weight 16 pounds. Cut by courtesy of editor.

---

**The Medical Commission** in charge of Saratoga Springs, has been abolished. Dr. Albert Warren Ferris, the Superintendent, has entered private practice at Saratoga Springs.

---

**Twilight Sleep** has been abandoned at Johns Hopkins Hospital, after a year's trial, on account of the danger, to both mother and child.

## Figures and Tables

**Figure f1:**
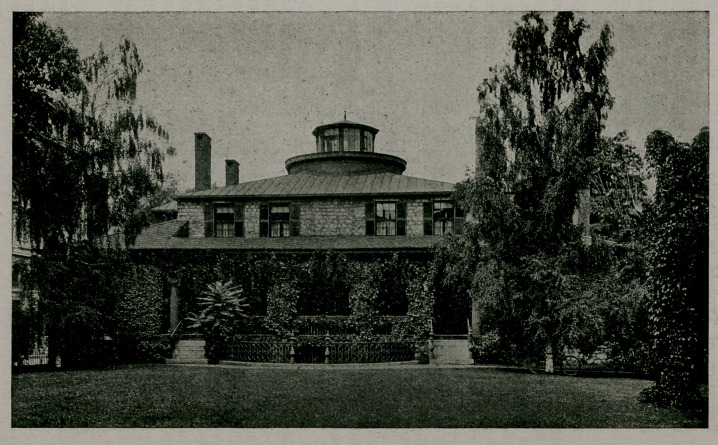


**Figure f2:**